# HIV Prevalence and Associated Factors among Foreign Brides from Burma in Yunnan Province, China

**DOI:** 10.1371/journal.pone.0115599

**Published:** 2014-12-23

**Authors:** Yin Xu, Li Ru Fu, Manhong Jia, Genyin Dai, Qing Wang, Peng Huang, Hui Zheng, Zhihang Peng, Lu Wang, Rongbin Yu, Ning Wang

**Affiliations:** 1 Department of Epidemiology and Biostatistics, School of Public Health, Nanjing Medical University, Nanjing, China; 2 Yunnan Provincial Center for Disease Control and Prevention, Kunming, China; 3 National Center for AIDS/STD Control and Prevention, Chinese Center for Diseases Control and Prevention, Beijing, China; UNC Project-China, China

## Abstract

**Background:**

Many Burmese women have migrated to Yunnan Province and married local residents over the past few decades; however, limited information is available on their HIV prevalence and ability to cope with HIV. This study aims to assess the prevalence of HIV and knowledge related to AIDS, as well as to discover possible risk factors of HIV infection among foreign brides from Burma in Yunnan Province.

**Methods:**

A cross-sectional study was taken of all Burmese cross-border wives residing in Tengchong County using standardized questionnaires. HIV and syphilis testing was conducted at the same time.

**Results:**

Among 600 Burmese brides, the HIV prevalence was 2.17%. Those aged 21–30, those with higher education levels and those who had resided in China less than one year had higher infection rates. The AIDS awareness rate of 39.50% was very low in this population. Only 28.67% of participants had ever been involved in prevention services. The rate of condom use was low. Classification by age, education, occupation, prior HIV testing and prior use of HIV prevention services showed a statistically significant association with mean knowledge score (p<0.05). Residing in China less than one year (OR = 3.86, 95% CI = 1.09–13.70) and having casual sex in the last year (OR = 10.49, 95% CI = 1.20–91.59) were risk factors for HIV infection.

**Conclusions:**

Burmese brides in China are not only exposed to a high risk of HIV infection, but also seriously lack response capabilities. Educational interventions and control efforts are practical approaches that need to be strengthened among this population.

## Introduction

Over the last two decades, China has witnessed an expanding epidemic of HIV/AIDS. The current national prevalence is about 0.058%, but the epidemic is more severe in some localized areas [1]. Yunnan, a province in southwest China with a high concentration of ethnic minorities and a long border with Burma, Laos and Vietnam, had the first Chinese HIV outbreak among injecting drug users (IDU) in 1989 and is still considered a key HIV epicenter [2], [3]. By the end of 2011, Yunnan had recorded a cumulative 93,567 cases of HIV infection, accounting for 12.0% of the total reported cases nationwide. In recent years, sexual transmission has overtaken IDU as the most commonly reported mode of HIV transmission in Yunnan Province [4]–[6].

For specific geographic and historical reasons, transnational marriages across the Chinese/Burmese border are common in Yunnan. Many Burmese women have migrated to Yunnan and married local residents. The main reason may be the imbalanced sex ratio in Yunnan: Due to a traditional preference for sons and pre-natal sex selection, a large number of so-called ‘surplus’ men or young, poor, unmarried men has emerged [7], which has resulted in an increasing demand for spouses from neighboring countries. In addition, Yunnan shares a similar culture and language along its border region with Burma, and relative to the situation in Burma, the Chinese economic level and social stability also has a certain appeal to Burmese border women. Burma is one of the Southeast Asian countries hardest hit by the HIV epidemic. The HIV prevalence among general adults (aged 15–49 years) in Burma in 2012 was estimated to be 0.60%, about ten times the rate in China [8]. Moreover, the region of Burma bordering Yunnan Province reported HIV infection rates significantly higher than the national average [9], [10]. Such an environment puts Burmese women at risk of HIV infection. Furthermore, some studies have indicated that cross-border movement may increase the risk of HIV transmission [11], [12]. The general prevalence of HIV in Tengchong County is 0.60%, but HIV testing conducted in Yunnan’s 25 border counties showed that the positive rates in the cross-border marriage population were as high as 1.64% [13]. One study discovered that cross-border female sex workers (FSWs) in Yunnan had a higher HIV infection rate than the local FSWs [14]. A survey conducted among Burmese-Chinese mixed couples in Tengchong County also showed that HIV prevalence among Burmese wives was 1.98%, while that of their Chinese husbands was 1.35% [15]. Another study reported that the HIV positive rates in Burmese brides and their Chinese husbands were 2.2% and 1.4%, respectively [16]. However, the majority of Burmese brides married to Chinese men in traditional folk weddings, without legal marriage registration, has resulted in a large number of de facto marriages. These women are therefore not subject to the protection and recognition of the law in China. Currently, there is no premarital examination and mandatory HIV screening among cross-border marriage couples in Yunnan border areas. These factors indicate that Burmese brides in Yunnan may have a higher risk of HIV infection than local residents. There are also the risks of mother to child transmission (MTCT) and transmission between spouses to consider. Burmese brides may be at risk of the HIV epidemic in China.

Accordingly, Burmese brides should be made a key target for intervention. In order to prevent and control AIDS more effectively, since 2011 foreign spouses can participate in the free national HIV/AIDS prevention and control policies, including free antiretroviral treatment. However, limited information is currently available on the epidemic situation of this unique population, as well as on their ability to cope with HIV. Thus, we conducted an epidemiologic and serological study to simultaneously identify the prevalence of HIV/AIDS among Burmese brides in Tengchong County and to assess their knowledge levels and risk behaviors related to HIV/AIDS. These findings are hoped to provide the necessary materials for follow-up interventions.

## Materials and Methods

### Ethics Statement

The study was approved by the ethical committee of Nanjing Medical University ("F", "CH", "Nanjing Med U", "FWA00001501", "NANJING", 11/21/2004). The objectives and procedure of the study and the potential risk and benefits of participation were given to potential participants during recruitment. Our study did not include minors/children. Written informed consents were obtained from all participants in the survey.

### Participates and questionnaire interview

This research adopted a cross-sectional study design. A census study of all Burmese cross-border wives residing in Tengchong County was conducted in September 2009. Burmese brides were defined as follows: (a) female at least 16 years of age; (b) Burmese nationality; (c) married to a local Chinese man, including those who did not have legal marriage registration but were de facto couples; and (d) living with their partners in Tengchong County. Study participants were recruited by local workers affiliated with Yunnan Provincial Center for Disease Control and Prevention. Local workers made household surveys first and counted the number of transnational marriages, including spouses who were undocumented immigrants. We summarized the data from their list to get the scale of transnational marriage in the county.

Face-to-face interviews were conducted one-on-one with trained staff using a structured questionnaire. The questionnaire (S1 Questionnaire) was designed specifically for Burmese brides and was based on the national and Yunnan Province’s HIV sentinel surveillance questionnaire. The content of the questionnaire included self-reported socio-demographic characteristics, HIV/AIDS-related knowledge, drug use, sexual behaviors, and HIV/AIDS-related prevention services. Participants were assured of confidentiality. Necessary assistance was provided to a few participants who had difficulty understanding either Mandarin Chinese or the local dialect.

### Serological Testing

Blood samples collected from each participant were transported to laboratories within 12 hours and screened for syphilis and HIV antibodies by enzyme-linked immunosorbent assay (ELISA; Beijing BGI-GBI Biotech Co., Ltd., China). Positive tests were confirmed at the Yunnan Provincial Center for Disease Control and Prevention (Yunnan CDC) using Western blot (WB; MP Diagnostics, Singapore).

### Statistical Analysis

Questionnaire data were double-entered and then checked for accuracy using EpiData software (V.3.1; The EpiData Association, Odense, Denmark). Descriptive analyses were conducted to elucidate the sociodemographic and behavioral characteristics of the participators. The chi-square (χ2) test and ANOVA analyses were used to conduct comparison tests. Categorical factors associated with HIV/AIDS infection were analyzed by univariate logistic regression, including baseline demographic and behavioral characteristics. Variables significant at a level of 0.2 in univariable analysis were fitted simultaneously into the multivariate model, and the standard for the variables to remain in the model was p<0.05. A multiple logistic regression model was constructed to select independent risk factors for HIV infection, while controlling for potential confounding factors. Both odds ratios (OR) and 95% confidence intervals (CI) were obtained for each explanatory variable in the final models. All statistical analyses were performed using Stata/SE (V.12.0 for Windows; StataCorp LP, College Station, USA), and a p value of less than 0.05 was considered to be statistically significant.

## Results

### Demographic characteristics and HIV prevalence

A total of 600 participants were studied and their mean age was 27.86±7.65 years old. The majority of participants were single and were engaged in agriculture before moving to China. About 71.00% of the Burmese brides were ages 30 or below, and only about 17.00% had attained a middle school education or higher. Only 10.17% (61/600) of participants had been tested for HIV/AIDS before moving to China, and 65.57% (40/61) of those knew the results.

Among all participants, 13 were anti-HIV antibody-positive and none was infected with syphilis at the time of study; the overall prevalence of the anti-HIV antibody-positive in this study population was 2.17% (13/600). There was great variation in HIV prevalence among different age groups and different education levels (Table 1). Prevalence was higher in the group aged 21–30 (3.53%) and in the group with higher education levels (4.90%). Significant difference was also observed for HIV prevalence among participants with different durations of residence in Yunnan (*p*<0.05); participants who had lived in Yunnan less than one year had a higher infection rate (4.78%). A total of 28.67% (172/600) of participants were involved in prevention services: 23.16% had received condoms promotion/HIV consultation and testing, and smaller percentages had accepted drug maintenance treatment/clean needles provision (2.50%) and peer education (6.50%). No association was found between HIV infection and receiving prevention services.

**Table 1:** Social demographic characteristics by HIV infection status.

Variables	HIV positive	HIV negative	Total N	*P* value
	(n = 13) N (%)	(n = 587) N (%)		
**Age (years)**				0.027
∼20	1(1.16)	85(98.84)	86	
21∼30	12(3.53)	328(96.47)	340	
31∼	0	174	174	
**Education level**				0.037
Primary school or below	8(1.61)	490(98.39)	498	
high school or above	5(4.90)	97(95.10)	102	
Marital status in Burma				0.068
Single	9(1.72)	515(98.28)	524	
**Married or Cohabiting**	1(7.14)	13(92.86)	14	
Divorced or Widowed	3(4.84)	59(95.16)	62	
durations of residence in Yunnan (year)				0.005
<1	9(4.78)	179(95.22)	188	
≥1	4(0.97)	408(99.03)	412	
**HIV test in Burma**				0.137
Yes	3(4.92)	58(95.08)	61	
No	10(1.86)	529(98.14)	539	
**Occupation in Burma**				0.355
Agricultural sector	11(2.03)	532(97.97)	543	
Others	2(3.51)	55(96.49)	57	
**Current occupation**				0.838
Agricultural sector	13(2.20)	579(97.80)	592	
Others	0	8	8	
**Condoms promotion/HIV consultation and testing**				0.501
Yes	2(1.43)	137(98.57)	139	
No	11(2.38)	450(97.62)	461	
**Drug maintenance treatment/Clean needles provision**				0.717
Yes	0	15	15	
No	13(2.22)	572(97.78)	585	
**Peer education**				0.414
Yes	0	39	39	
No	13(2.32)	548(97.68)	561	

### Knowledge of HIV/AIDS

HIV/AIDS knowledge was assessed by eight items. Each question was assigned a score: a correct answer was recorded as 1 point, while wrong or “don't know” were 0 points. The comprehensive correct rate of HIV/STD knowledge was only 18.17% (109/600) and the rate of AIDS awareness (achieved six or more points) was 39.50% (237/600), while 28.17% (169/600) of participants achieved 0 points. Questions with an incorrect response rate higher than 50% are as follows: “Mosquito bites can spread HIV” (61.67%); “Having meals with AIDS patients can result in HIV infection” (53.83%); and “Using condoms properly can’t reduce HIV transmission” (56.00%).

Table 2 summarizes the mean score and P values for knowledge of HIV/AIDS among Burmese brides. Classification by age, education, occupation, history of HIV test in Burma and high risk sexual behaviors (commercial sex or casual sex) showed a statistically significant association with mean knowledge score (*p*<0.05). Those aged between 21 and 30, those who had undergone HIV testing in Burma and those who had commercial sex or casual sex in the last year had a higher mean knowledge score; however, Burmese brides who were previously agricultural workers in Burma or who had lower education levels had a lower mean knowledge score. Simultaneously, participants who had ever received HIV prevention services (peer education, drug maintenance treatment/clean needles provision and condoms promotion/HIV consultation and test) had a higher mean knowledge score.

**Table 2:** Mean Knowledge Score by demographic and selected variables.

Variables	Total N	Means	Std	*P* value
**Age (years)**				0.003
∼20	86	3.30	3.28	
21∼30	340	4.33	3.03	
31∼	174	3.58	2.95	
**Education level**				< 0.001
Primary school or below	498	3.61	3.04	
Middle school or above	102	5.71	2.58	
**Marital status in Burma**				0.194
Single	524	4.01	3.07	
Married or Cohabiting	14	4.71	2.89	
Divorced or Widowed	62	3.37	3.03	
**Durations of residence in Yunnan (year)**				0.134
<1	188	4.03	3.24	
≥1	412	3.93	2.92	
**HIV test in Burma**				0.001
Yes	61	5.23	2.67	
No	539	3.82	3.08	
**Occupation in Burma**				< 0.001
Agricultural sector	543	3.81	3.06	
Others	57	5.42	2.81	
Drug abuse				0.349
Yes	2	6.00	0.00	
No	598	3.95	3.07	
**Having commercial sex**				0.030
Yes	5	7.00	2.23	
No	556	3.98	3.09	
**Having casual sex**				0.010
Yes	6	7.16	2.04	
No	584	3.91	3.08	
**Peer education**				< 0.001
Yes	39	6.07	2.51	
No	561	3.82	3.05	
**Condoms promotion/HIV consultation and testing**				< 0.001
Yes	139	5.07	2.46	
No	461	3.62	3.16	
**Drug maintenance treatment/Clean needles provision**				0.030
Yes	15	5.67	2.47	
No	585	3.92	3.07	

### Sexual behavior and drug use

A total of two respondents had ever taken drugs, while none had a history of intravenous drug use; none of those who had taken drugs were infected with HIV. Most of the participants reported having sexual intercourse in the last 12 months. A total of 90.16% (541/600) had sex with husbands, but their condom usage rate was poor. A total of 90.20% (488/541) of participants preferred not to use condoms. There were low rates of infection among participants who had sex with husbands using a condom every time, while the highest rate of infection was found among those who never used a condom. (Table 3) Five participants reported having commercial sex, and six had casual sex with no condom use each time. We analyzed the influencing factors of high risk sexual behaviors (defined as having commercial or casual sexual intercourse with inconsistent condom use) and discovered that people who scored six or greater on the knowledge score were more likely to engage in high risk sexual behaviors (OR  = 2.26, 95% CI  = 1.07–4.79).

**Table 3:** Risk factors and their associations with HIV infection among Burmese brides.

Variables	Total	HIV positive	OR (95% CI)	*P* value
	N	N (%)		
**Age (years)**				
∼20	86	1(1.16)	1	
21∼30	340	12(3.53)	3.11(0.40–24.25)	0.279
31∼	174	0	–	–
**Education level**				
Primary school or below	498	8(1.61)	1	
Middle school or above	102	5(4.90)	3.16(1.11–9.85)	0.040
**Duration of residence in Yunnan (year)**				
≥1	412	4(0.97)	1	
<1	188	9(4.78)	5.13(1.56–16.87)	0.007
**Knowledge score**				
<6	363	7(1.93)	1	
≥6	237	6(2.53)	0.75(0.25–2.28)	0.621
**Drug abuse**				
Yes	2	0	1	
No	598	13(2.17)	–	–
**Sex with husband**				
No	34	2(5.88)	1	
Yes	541	11(2.03)	0.33(0.071–1.56)	0.163
No answer	25	0		
**Condom use with husband**				
Never	488	10(2.05)	1	
Sometimes	43	1(2.33)	0.85(0.11–6.85)	0.883
Every time	10	0	–	–
**Commercial sex**				
No	556	12(2.16)	1	
Yes	5	1(20.00)	11.33(1.17–109.23)	0.036
No answer	39	0		
**Casual sex**				
No	555	11(1.88)	1	
Yes	6	2(33.33)	24.72(4.09–149.48)	< 0.001
No answer	39	0		

### Risk factors of HIV positivity

The factors strongly associated with HIV infection in univariable analysis (Table 3) were education level, duration of stay in China, and history of commercial sex and casual sex, while age, knowledge score, and having sex with a husband were not correlated with HIV infection. Variables significant (*p*<0.2) in the univariable analysis were considered for further evaluation in multivariable analysis, The multivariable model (Table 4) revealed that after controlling for age, the factors residing in China less than one year (OR = 3.86, 95% CI = 1.09–13.70), having casual sex in the last year (OR = 10.49, 95% CI = 1.20–91.59) increased the risk of HIV infection.

**Table 4:** Multivariate logistic regression analysis of risk factors associated with HIV positivity.

Variables	OR (95% CI)	*P* value
**Age(years)**		
21∼30 *vs.* <20	4.89(0.58–40.81)	0.142
**Education level**		
Middle school or above *vs.* Primary school or below	1.19(0.29–4.99)	0.805
**Residence time (year)**		
<1 *vs.* ≥1	3.86(1.10–13.70)	0.037
**Sex with husband(yes vs. no)**	0.53(0.15–1.92)	0.335
**Commercial sex(yes *vs.* no)**	9.52(0.98–116.29)	0.078
**Casual sex(yes *vs.* no)**	10.49(1.20–91.59)	0.034

## Discussion

Because of their mobility, proximity to an international border and the nature of their international marriages, cross-border brides are subject to various influences from peers, family and society, solidifying their status as a special population with a high risk of HIV infection in Yunnan Province. Some reports indicated that foreign brides in China may be a developing infection pool for AIDS, but quantitative studies on this topic are scarce. Our study investigated a sample of 600 Burmese brides aged 18–60 years and collected data on their knowledge and risk behaviors of HIV/AIDS, confirming this population’s vulnerability to HIV infection. Moreover, our study has substantial value in supporting effective and directed policies toward controlling HIV prevalence for this target population in Yunnan.

In our study the HIV prevalence among Burmese brides was 2.17%, higher than the general prevalence of 0.60% in local residents in Tengchong County and more than 38 times higher than the official HIV prevalence estimate of 0.058% among the general Chinese population [8]. This high infection rate may be due to several reasons. Because the HIV/AIDS infection rate of the general population in Burma is significantly higher than in China, these Burmese brides may be arriving from a higher infection pool and in some cases could be carrying premarital infection [17]. However, only 10.16% of these participants had premarital HIV testing in Burma, which inevitably adds to the risk of transmission. Secondly, the vast majority of Burmese brides in our study were younger (aged 21–30) with higher levels of sexual activity, and in some cases may have married Chinese residents through illicit channels [18], [19]. Coupled with the unstable environment of migration, these brides may have been exposed to a higher risk of HIV infection. This may somewhat explain why those aged 21–30 and those who had resided in China less than one year had higher infection rates in this study. Finally, there is a risk of transmission between couples. A meta-analysis showed that the overall estimate of HIV prevalence through heterosexual transmission in discordant couples is 17% in Yunnan province [20]. One study of 5742 Chinese-Burmese mixed couples in Yunnan found that 3.1% of the couples were HIV-infected discordant [21]. Uninfected brides may face the potential risk of secondary HIV transmission.

In recent years, epidemiological synergy has been discovered between HIV and syphilis. HIV and syphilis are both transmitted sexually and co-infection is common [22]. However, in our study, none of the participants were infected with syphilis at the time of study, which is very intriguing. We know that having multiple sex partners may be the main risk factor for infection [23]. While Burmese brides in our study were generally engaged in legitimate occupations and their marital statuses were relatively stable, most families had their own biological children. Cases that were HIV-positive but syphilis negative have also been reported in other studies. One study that detected syphilis among 11,368 HIV positive patients only found 151 cases of active syphilis (1.33%) [24]. Moreover, controversy has surrounded the area of HIV and syphilis co-infection; the interrelationship between them is complex and remains incompletely understood [25].

The AIDS awareness rate of 39.50% among foreign brides was distinctly lower than among rural residents in Yunnan (83.90%). Foreign brides’ lower levels of knowledge about AIDS may be related to their lower education. We found that brides with higher education levels had better mean knowledge scores, which may be explained by those groups having more chances to attend local AIDS awareness activities and being more willing to participate. This in turn may stimulate a desire to acquire more information pertaining to the disease. Similarly, the population involved in prevention and care programs in China had a higher knowledge score. This suggests the positive impact of community intervention and the necessity of promoting the government’s health services. However, only 28.67% of participants were involved in prevention services; coverage of these public services among foreign brides is considerably lacking. There was no direct correlation between AIDS awareness and HIV infection in this study, although those participants who were aware of AIDS (with higher scores) were more likely to engage in high risk sexual behaviors. This cognitively-dissonant “separation between knowledge and action” phenomenon has also been noted in other studies [26], [27]. It indicates that the process from knowledge acceptance to behavioral change is complicated, and some populations may need further intervention to encourage behavioral change.

The risk of HIV infection among foreign brides residing in China less than one year is 3.86 times that of brides who resided one year or more. This may be because those living in China less than one year were relatively younger, with higher levels of sexual activity and thus a higher risk of HIV infection. High risk sexual behaviors were found to be another risk factor of HIV infection in our study, which is consistent with many other studies [15], [28]. Those with casual sex partners were likely to have higher rates of partner change and low rates of condom usage, thus increasing their risk of contracting HIV from an infected partner.

Some limitations should be noted in considering the findings of our study. We did not conduct a questionnaire survey of foreign brides’ Chinese spouses simultaneously. If compared with the results from this study, more comprehensive and meaningful information might have been obtained. We hope this may be attempted in our future research. In addition, the sample size was not large, which makes the OR values unstable. Due to the study’s dependence on the questionnaire, some questions involved issues of the past such as condom usage. If respondents couldn’t remember clearly, this may have resulted in recall bias. Meanwhile, information bias may have limited participants’ ability to provide accurate information on sensitive issues such as sexual behaviors, resulting in an underestimation of the risk association. The use of a cross-sectional study design limited our ability to establish or analyze a causal relationship between HIV infection and risk factors. Moreover, the data was collected in 2009 and some elements of this population may have altered since then. However, given the scarcity of data on this population, this study nevertheless provides important baseline data for monitoring changes in future studies.

Considering the high prevalence of HIV, the low coverage of prevention services and the unique characteristics of Burmese brides in China, more effort is needed to control the HIV epidemic in this population [29]. Government or health institutions should strive to promote premarital HIV testing and other appropriate medical examinations in cross-border marriage registration. Additionally, the intensity and breadth of AIDS education should be actively strengthened in the border areas in order to minimize high risk sexual behaviors.

## Supporting Information

S1 Data (XLS)

S1 Questionnaire (DOC)
