# Supplementary material for: HIV Prevalence and Associated Factors among Foreign Brides from Burma in Yunnan Province, China
Source: PLoS One. 2014 Dec 23;9(12):e115599. doi: 10.1371/journal.pone.0115599 (PMC4275213; doi:10.1371/journal.pone.0115599)
Supplement: S1 Questionnaire — (DOC) [file pone.0115599.s002.doc]

**缅甸籍新娘健康调查问卷**

A01调查地点 市 县（市、区） 乡镇

A03调查所在地行政区划国标码 □□□□□□

A05问卷编号 □□□（001—999）

A06调查日期 □□□□年□□月□□日

你好，我叫……，来自……。我们正在进行一项调查，目的是了解人们对一些健康问题的知识和行为状况。请放心，本次调查不记名，我们会对你的回答保密。我们希望你的回答是你个人的真实情况。调查大约会占用你10分钟时间，调查结束时我可以为你提供一些帮助（例如你可以咨询一些健康方面的问题，我会尽量解答）。希望你支持我们的工作。谢谢！

询问调查对象：请问你最近是否参加过此项调查？若回答“是”则结束此次访问。

B01姓名

B02身份证号

B03 原籍（国家） ①缅甸 ②中国

B04出生年 年

B05文化程度 ①文盲 ②小学 ③初中 ④高中或中专 ⑤大专及以上

C01 一个感染了艾滋病病毒的人能从外表上看出来吗？ ①能 ②不能 ③不知道

C02 蚊虫叮咬会传播艾滋病吗？ ①会 ②不会 ③不知道

C03 与艾滋病病毒感染者或病人一起吃饭会感染艾滋病吗？ ①会 ②不会 ③不知道

C04 输入带有艾滋病病毒的血液会得艾滋病吗？ ①会 ②不会 ③不知道

C05 与艾滋病病毒感染者共用注射器有可能得艾滋病吗？ ①可能 ②不可能 ③不知道

C06 感染艾滋病病毒的妇女生下的小孩有可能得艾滋病吗？ ①可能 ②不可能 ③不知道

C07 正确使用安全套可以减少艾滋病的传播吗？ ①可以 ②不可以 ③不知道

C08 只与一个性伴发生性行为可以减少艾滋病的传播吗？ ①可以 ②不可以 ③不知道

D01 在原籍地您从事何种职业

1. 农民 ②工人 ③个体 ④餐饮及服务业 ⑤其它

D02 您来本地以前的婚姻状况 ①未婚 ②在婚 ③同居 ④离异或丧偶

D02-1 如果是丧偶，您的配偶是死于何种原因？

D03 您来本地以前是否接受过艾滋病检测？ ①有 ②无

D04 如果做过检测，是否知道结果？ ①知道 ②不知道

E01 您何时来到本地 □□□□年□□月

E02 您现在从事的职业

①农民 ②工人 ③个体 ④餐饮及服务业 ⑤其它

F01最近一年，您与配偶发生过性关系吗？ ①有 ②无

F02你最近一次与配偶发生性行为时使用安全套了吗？ ①是 ②否 ③拒答

F03您最近12个月您与配偶发生性行为时使用安全套的频率如何？

1. 从未使用 ②有时使用 ③每次都用 ④拒答

G01最近一年，您与商业性伴发生过性行为吗？ ①是 ②否 ③拒答

G02您最近一次与商业性伴发生性行为时使用安全套了吗？ ①是 ②否 ③拒答

G03最近一年，你与商业性伴发生性行为时使用安全套的频率如何？

①从未使用 ②有时使用 ③每次都用 ④拒答

H01最近一年，您与临时性伴发生过性行为吗？（临时性伴是指非商业非固定性伴，即偶尔有性行为非商业性的异性性伴，如一夜情等。） ①是 ②否 ③拒答

H02您最近一次与临时性伴发生性行为时使用安全套了吗？ ①是 ②否 ③拒答

H03最近一年，你与临时性伴发生性行为时使用安全套的频率如何？

1. 从未使用 ②有时使用 ③每次都用 ④拒答

I01你吸毒吗？ ①是 ②否 ③拒答

I02 您吸毒方式是什么 ①口吸 ②静脉注射

I03你注射过毒品时，是否与别人共用过针具？ ①是 ②否（跳至I01） ③拒答

I04最近六个月注射毒品时，你与别人共用针具的频率如何？

1. 从未共用过 ②有时共用 ③每次都共用 ④拒答

J01 您有接受过安全套宣传和发放、艾滋病咨询与检测吗？ ①有 ②无

J02 您有接受过社区药物维持治疗/清洁针具提供或交换吗？ ①有 ②无

J03 您有参加过同伴教育吗？ ①有 ②无

K01最近一年，您是否做过艾滋病检测？  ①是 ②否 ③拒答

K02 您知道自己的检测结果吗？ ①是 ②否 ③拒答

调查到此结束，谢谢你的合作。为了解你的健康状况，我们需要对你采血，进行梅毒和艾滋病检测。

L01本次调查是否采血 ①是（跳至T03） ②否

L02本次调查未采血原因 ①既往检测HIV抗体阳性 ②拒绝采血

L03 HIV抗体检测结果 第一次ELISA初筛 ①阳性 ②阴性（跳至T04）

第二次ELISA复检 ①阳性 ②阴性

确认试验 ①阳性 ②阴性 ③可疑 ④未检测

L04梅毒检测结果 ①阳性 ②阴性

调查员签字 督导员签字
